# The Association between Intertwin Difference in First Trimester Crown-Rump Length, Nuchal Translucency, and Birth Weight Discordance in Twin Pregnancies: A Retrospective Cohort Study

**DOI:** 10.1155/2022/6539038

**Published:** 2022-11-17

**Authors:** Zachary Michael Ferraro, Tinghua Zhang, Felipe Moretti, Karen Fung-Kee-Fung

**Affiliations:** ^1^Faculty of Medicine, University of Ottawa, 451 Smyth Rd, Ottawa, Ontario, Canada K1H 8M5; ^2^Department of Obstetrics and Gynaecology, Faculty of Medicine, University of Toronto, 123 Edward Street, Toronto, Ontario, Canada M5G 1E2; ^3^The Ottawa Hospital Research Institute, 1053 Carling Ave, Ottawa, Ontario, Canada K1Y 4E9; ^4^Department of Obstetrics, Gynecology and Newborn Care, Division of Maternal-Fetal Medicine, University of Ottawa, The Ottawa Hospital, 501 Smyth Rd, Ottawa, Ontario, Canada K1H 8L6

## Abstract

**Background:**

Discordant birth weight in twins is linked to poor outcomes and predicting this discrepancy may lead to enhanced screening and surveillance. Our purpose was to quantify the relationship between intertwin nuchal translucency (NT) and crown-rump length (CRL) discordance with birth weight discrepancies ≥ 20%.

**Methods:**

We conducted a retrospective cohort study of 887 live twin births delivering at a Canadian tertiary care center over a 7-year period who opted for integrated prenatal screening. Categorical data are presented as numbers and percentages, and continuous data are expressed as means and standard deviations. Chi-square tests, Fisher's Exact tests, or *T*-test were performed as appropriate. We then used published data and receiver operating curves to determine the optimal thresholds for predicting birth weight discordance based on first trimester intertwin NT differences. These values were used in multivariate logistic regression models accounting for known confounders.

**Results:**

Roughly 16% of twin pairs exhibited ≥ 20% difference in birth weight. Twin pairs with a CRL discordance greater than 10% have nearly a 4 times greater likelihood of having a birth weight discordance greater than 20% (OR 3.71, CI 2.24-6.14) while controlling for chorionicity, maternal age, gestational age at delivery, maternal body mass index (BMI), and parity. In these models, intertwin NT discordance ≥ 20% (OR 1.16, CI 0.77-1.77) and NT discordance ≥ 14% (OR 1.08, CI 0.73-1.60) were not statistically significant predictors of twin birth weight differences. However, when evaluating the effect of the larger intertwin NT value corresponding to the 95^th^ percentile, an NT difference ≥ 0.9 mm was predictive of birth weight discordance ≥ 20% (OR 2.53, CI 1.21-5.29).

**Conclusion:**

Although intertwin CRL and NT discordance measured via ultrasound between 11-14 weeks gestation are related to birth weight discordance, there is uncertainty as to whether twin birth weight differences are related to adverse pregnancy outcomes in this population.

## 1. Introduction

The incidence of twin pregnancy has increased over the past several decades due to delayed childbearing and increased use of new reproductive technologies [1]. Currently, twins comprise approximately 3% of all births [2]. Despite this relatively low incidence, twins, especially monochorionic twins, are disproportionately represented in perinatal morbidity and mortality figures due to preterm birth (50%) and fetal growth problems (15%) [3, 4]. Fetal outcomes appear to be worse when intertwin birth weight differences exceed 20% [5]. Early prediction of twins likely to experience growth issues may lead to improved outcomes through enhanced personalized surveillance and appropriate patient counselling to set realistic parental expectations [6].

During the first trimester differences between cotwins in fetal measurements made on ultrasound examination are suggested to be early markers of twin complications including discordant fetal growth [7]. At present, no studies have explicitly looked at the relationship between intertwin crown-rump length (CRL) and nuchal translucency (NT) discordance with twin birth weight differences, and conflicting results exist with respect to the utility of NT and CRL as early predictors of adverse neonatal outcomes. Kagan et al. [8] completed an analysis of a retrospective birth cohort that measured NT at 11 to 13 + 6 weeks in 512 monochorionic twin pregnancies and showed that discordance in NT of ≥ 20% is found in 25% of monochorionic twins. In this group, the risk of early fetal death or development of severe twin-twin transfusion syndrome (TTTS) was more than 30%. Encouragingly, if the discordance was less than 20% the risk of complications was less than 10% when NT measures are concordant in the first trimester. Lewi et al. [9] reported that differences in CRL predicted TTTS, severe discordant growth, or intrauterine death in a prospective cohort of 202 monochorionic diamniotic (MCDA) twin pregnancies. Zipori et al. [10] studied 89 MCDA twin pregnancies and constructed receiver operating curves (ROC) to determine the ideal intertwin NT discordance cut points. They concluded that NT discordance > 23.7% had high a negative predictive value (86.8%) but a positive predictive value of only 52.4% for the discrimination of a composite score of adverse fetal outcomes that included death of one or both fetuses, development of TTTS, the presence of fetal anomalies, estimated fetal weight discordance > 25% at 28-week ultrasound, or birth weight discordance > 25%. In a multicenter retrospective cohort study, Allaf et al. [11] reported that NT, CRL, and combined discordances in MCDA twin pregnancies were not predictive of adverse composite obstetric and neonatal outcomes. Despite the heterogeneity in both methodology and measured outcomes for the aforementioned studies, fetal CRL and NT measurement at 11-14 weeks' gestation continues to be scrutinized as a screening method used to predict diverse pregnancy outcomes including aneuploidy, cardiac abnormalities, genetic syndromes, and TTTS [9–13].

In our region NT measurement, as part of early aneuploidy screening, is offered to all pregnant women as part of standard obstetrical care. As opposed to looking specifically at loss rates or adverse outcome data as has been reported previously [7–13], we aimed to evaluate the impact of NT and CRL discordance in twins on live births with a specific focus on intertwin growth discrepancies as this tends to proceed adverse outcomes. Thus, our primary objective was to determine whether intertwin CRL and NT discordance measured via ultrasound at 11-14 weeks gestation is associated with birth weight discordance. Secondary objectives aimed to use multivariable models to quantify if NT and CRL discordance can be used to make clinically meaningful prediction of birth weight discrepancy.

## 2. Methods

We conducted a retrospective cohort study of all live twin births delivering over a 7-year period who opted for first trimester screening. To better understand the relationship between CRL and NT discordance and birth weight discrepancies > 20%, we collected data on 887 patients with monochorionic-monoamniotic (MCMA), MCDA, and dichorionic-diamniotic (DCDA) twin pregnancies from a tertiary care center in Canada. Data were extracted from the electronic medical and birth records of mothers and their twin offspring. Exclusion criteria included fetuses with documented chromosomal abnormalities, structural anomalies, intrauterine death of a cotwin, TTTS, delayed interval delivery of a second twin, and pregnancies of higher fetal order with a monochorionic twin. We included twins of all types of placentation (DCDA, MCDA, and MCMA) in our analysis. DCDA twins were included while they too may exhibit significant growth discordance although the clinical implications and sequelae of discordant growth in this twin group can differ from those of the MC population [14]. We aimed to explore the association between CRL and NT discordance and growth discordance in healthy twins who met our exclusion criteria. Gestational age was determined by the CRL of the fetuses at the 11–14-week scan. Chorionicity was assigned by first trimester ultrasound evaluation according to the number of placentae and the presence of the lambda or T-sign and was confirmed after birth by pathology. The technique used to measure NT is described by Nicolaides et al. [15] and that used to quantify CRL is reviewed in our national specialty society clinical practice guideline [16]. All CRL and NT measurements were performed by qualified sonographers certified by the Fetal Medicine Foundation, the American Registry of Diagnostic Medical Sonography, and within an imaging unit accredited by the American Institute of Ultrasound in Medicine.

Measurements of the CRL and NT were taken between 11‐13 weeks + 6 days gestation as determined by ultrasound. NT discordance (%) was calculated as 100 × (larger NT − smaller NT)/larger NT. An NT difference of > 20% was considered clinically meaningful and based on previously published literature [8, 11]. Next receiver operating curves were constructed (data not shown). It was determined that 14% NT discordance was the optimal cutoff value for our study population when evaluating twin birth weight discordance as the primary outcome; a difference likely related to the homogeneity of our cohort. Lastly, to ascertain if the larger NT was driving the observed relationship, we established that the difference in NT between twins that corresponded to the 95^th^ percentile was equal to 0.9 mm. CRL discordance (%) was calculated as 100 × (larger CRL − smaller CRL)/larger CRL. For purposes of our analysis, a CRL discordance of > 10% is considered clinically meaningful, based on previously published literature [15]. The primary outcome of fetal birth weight growth discordance > 20% was obtained using absolute change in birth weight between cotwins. This threshold was chosen based on national guidelines that recommend close follow up if such an intertwin difference is observed [17] as it has been previously linked to poor outcomes [5, 6]. Bivariable assessment was completed to examine the relationship between CRL and NT discordance and intertwin birth weight differences. Then, differences in CRL and NT (independent variables) were fixed as a continuous absolute predictor of growth discordance using regression with dichotomized or linear outcomes where appropriate. Lastly, we ran multivariate probability models that included factors known to be related to birth weight.

We first compared baseline maternal characteristics and fetal outcomes in live twins by group (intertwin birth weight differences less than 20% vs. greater 20%). Categorical data are presented as numbers and percentages, whereas continuous data are expressed as means and standard deviations. Chi-square tests, Fisher's Exact tests, or *T*-test was performed as appropriate. Further multivariable logistic regression was used to examine the effect of NT discordance on intertwin birth weight difference, adjusting for clinically relevant covariates including chorionicity, maternal age, gestational age, maternal pregravid body mass index (BMI), and parity. We reported odds ratio (OR) and 95% confidence intervals (CI). All analyses were conducted using SAS version 9.3 (SAS Institute Inc). All statistical tests were 2-sided. The study protocol was approved by the institutional research ethics board (study protocol number REB20140143-01H).

## 3. Results

**Figure 1:**
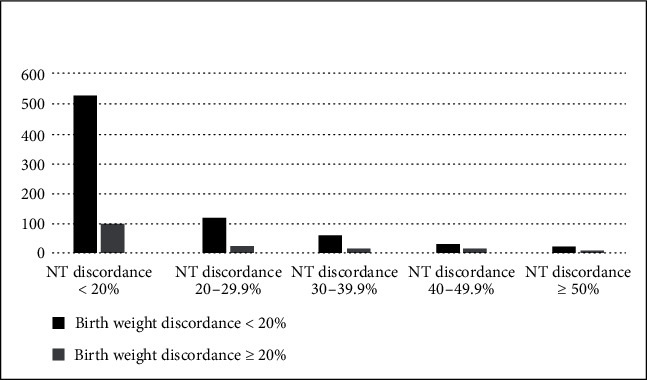
Nuchal translucency and birth weight discordance distribution.

**Figure 2:**
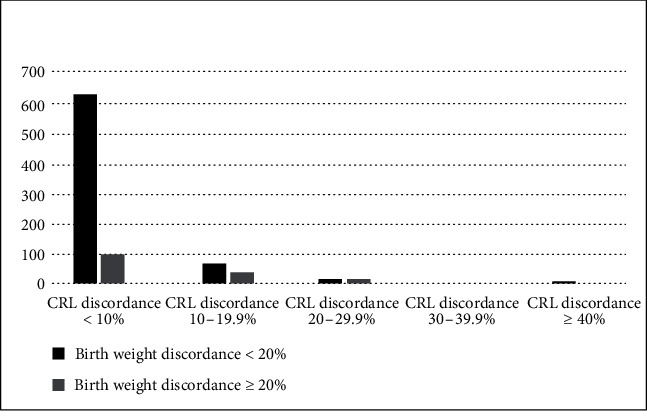
Crown rump length and birth weight discordance distribution.

In our retrospective study complete data was available for a total of 887 healthy, live twin pregnancies. Maternal characteristics at baseline, including age, BMI, IVF, fetal sex distribution, chorionicity, and parity, were not different between groups when categorized according to ≥20% birth weight discordance (Table 1). Eighty four percent of twin pairs had birth weight discordance < 20%. However, the CRL difference (2.6 mm vs. 4.5 mm) and the birth weight difference (204 g vs. 732 g) between twins were larger, and the gestational age at delivery shorter (35.8 weeks vs. 34.7 weeks) when birth weight discordance was ≥20% (Table 1). Independent of birth weight discordance, most twin pairs had NT discordance < 20% (Figure 1) and CRL discordance < 10% (Figure 2).

Prior to multivariable modeling we performed a collinearity diagnostic test that showed no collinearity among variables in our models. A CRL discordance of > 10% is considered clinically meaningful [17] and was used as our independent variable in regression models predicting birth weight discordance (Table 2). In multivariable analysis adjusting for chorionicity, maternal age, gestational age at delivery, maternal pregravid BMI, and parity, only CRL discordance > 10% and gestational age at delivery were statistically significant. For instance, twins with CRL discordance greater than or equal to 10% have 3.7 times the odds of having a birth weight discordance ≥ 20% compared to twins with CRL discordance < 20% while controlling for potential confounders. Whereas, the odds of having a weight discordance ≥ 20% decrease 12.7% for every 1 week increase in gestational age at delivery (Table 2).

With respect to evaluating the effect of NT on twin birth weight discordance, we first based the independent variable cutoff value on previous literature [8, 11] as an NT difference of > 20% was cited as clinically meaningful. Next, using receiver operating curves, it was determined that 14% NT discordance was the optimal cutoff value in our population when evaluating birth weight discordance. Interestingly, in both models (NT difference > 20% and > 14%) while controlling for chorionicity, maternal age, maternal pregravid BMI, and parity, again, only gestational age at delivery was statistically significant. The odds of having a weight discordance ≥ 20% decreased 12.7% for every 1 week increase in gestational age at delivery (Tables 3 and 4). Our final predictive model was used to ascertain if the larger NT between twin pairs was driving the observed relationship in birth weight discordant twins. We established that the difference in NT between twins that corresponded to the 95^th^ percentile was equal to 0.9 mm, and this value was used in multivariable analysis. When the intertwin NT difference was greater than 0.9 mm, there was a 2.5 greater likelihood that birth weight discordance would be greater than 20% (OR 2.53, CI 1.21-5.29). Of note, the relationship between gestational age at delivery was also statistically significant (Table 5).

## 4. Discussion

In this single-centered retrospective Canadian twin cohort, we evaluated the effect of first trimester intertwin NT and CRL differences measured via sonography on birth weight discordance given its reported relationship with neonatal morbidity and mortality. Using strict inclusion and exclusion criteria, we aimed to determine if abnormal growth in fetuses without anomalies or TTTS can be detected early in trimester one. In this highly selective healthy live-twin population, nearly 1 in 5 twin pairs exhibited ≥ 20% difference in birth weight. In line with our hypotheses, intertwin CRL difference greater than 10%, NT discordance (> 20% and > 14%), and absolute NT difference greater than 0.9 mm (corresponding to the 95^th^ percentile) were associated with twin birth weight discrepancy. Furthermore, we show that CRL discordance greater than 10% and an absolute NT difference corresponding to the 95^th^ percentile are associated with an increased likelihood of twin birth weight discordance greater than 20%.

Understanding first trimester sonographic features of fetal growth discordance in twins may help predict birth weight discordance [18], selective IUGR, and TTTS [19, 20]. In 1993, Achiron and Blickstein [21] were the first to report a case of an IVF twin pregnancy where first trimester CRL discordance between 7-11 weeks preceded birth weight difference of 26%. In our study, IVF pregnancy was equal between groups and unrelated to the primary outcome. Our results, however, are similar to those reported by Dias et al. [22]. In a retrospective cohort of 506 dichorionic and 154 monochorionic twin pregnancies, they demonstrated that CRL discordance was related to subsequent pregnancy loss and birth weight discordance; and CRL discrepancy was independent of chorionicity in their twin cohort. We report similar findings using regression analysis suggesting that the chorionicity measured via first trimester ultrasound (and confirmed with pathology) was unrelated to our primary outcome of twin birth weight discordance. It is speculated that the difference in twin CRL at 11-14 weeks is likely to represent physiological variation in most cases [22]. In further support of our findings, Weissmann-Brenner et al. [18] showed that discordance in first trimester CRL measurement in MC and DC twins can predict discordance of more than 25% in neonatal birth weight. We add to the current literature and report findings from a larger sample of twins that focused exclusively on intertwin birth weight discordance in monochorionic and dichorionic gestations without TTTS to ascertain, in a healthy cohort, the predictive value of first trimester NT and CRL differences. Furthermore, the length of gestation (35.8 vs. 34.7 weeks) and mean birth weight (2445 g vs. 2131 g) was significantly greater in twins with birth weight discordance < 20% compared to their counterparts with birth weight discordance ≥ 20%. Reasons for this observation may include spontaneous preterm birth and early sonographic recognition of fetal growth discrepancies during routine checkups leading to iatrogenic delivery secondary to earlier intervention.

While not the a priori purpose of our paper, several studies have reported on the effect of discrepant NT and CRL on twin pregnancy outcomes that warrant discussion [11, 23, 24]. Both D'Antonio et al. [24] and Allaf et al. [11] reported that CRL is of poor predictive value for adverse perinatal outcome twin pregnancies. D'Antonio et al. [24] reported findings in partial agreement to ours from a retrospective cohort of 2155 twin pregnancies over a 10-year period. Using ROC curves and logistic regression, they showed that while monochorionicity was associated with double the risk of fetal loss, both CRL and NT discordance were poor predictors and not associated with risk of perinatal loss, respectively. Similarly, Allaf et al. [11] reported that NT, CRL, and combined discordances in MCDA twin pregnancies were not predictive of adverse composite obstetric and neonatal outcomes. In their multicenter trial of 9 perinatal centers in the United States, they obtained data from 177 MCDA pregnancies and observed a high (31%) overall risk of adverse composite obstetric outcomes, including intertwin growth discordance ≥ 20% in 8% of pregnancies [11]. Our prevalence of intertwin growth discordance ≥ 20% is higher than previously reported likely reflecting the inclusion of all twin types in our study and regional referral patterns to our center as the sole provider of tertiary level perinatal care. These findings have since been replicated by Litwinska et al. [23] in a large retrospective analysis of 6225 monochorionic and dichorionic twin pregnancies undergoing routine ultrasound examination at 11-13 weeks' gestation. They highlight that although CRL discordance is associated with an increased risk of fetal death, preterm birth, small for gestational age, and birth weight discordance greater than 20%, it was shown to be a poor screening test for adverse pregnancy outcome [23]. Similar results were reported by Cimpoca et al. [25] who concluded in MCDA twin pregnancies that NT measurement in the first trimester was a poor screening test for adverse pregnancy outcomes. They did suggest, however, that in twin pairs when one or both NT values was ≥ 95^th^ percentile, it was associated with an increased risk of fetal loss or need for endoscopic laser surgery less than 20 weeks gestation.

With respect to chorionicity, when established with ultrasound, our cohort included 174 MCDA twins, of which 25/174 (14%) of these twins had birth weight discordance ≥ 20%. When chorionicity was verified with pathology, we showed that in a total of 143 MCDA twin pairs, 23/143 (16%) of these had birth weight discordance ≥ 20%. The small discrepancy in assignment of chorionicity between methodologies may reflect, in part, different denominators in the groups. In some instances, chorionicity was established solely by pathological examination of the placenta (the gold standard), whereas others only were established by ultrasound if the placenta sample was not available for examination. Despite these findings, it is important to note that chorionicity was not significantly different between birth weight groups (< 20 vs. ≥ 20) in our study and was unrelated to the primary outcome of birth weight discordance in multivariate analysis.

A limitation of our study is the retrospective nature of the analysis which precludes causal inferences from being made and the fact that the data came from a single tertiary care center which limits generalizability. The strengths of our study include a large sample size and single center nature of data collection and clinical care. All twins in our dataset with significant growth discordance were cared for by the same group high-risk specialists which increases the internal validity of the results. At our institution, we have strict quality assurance procedures in place with respect to credentialing sonographers and sonologists performing the thousands of NT measurements at our institution annually. Similarly, with respect to birth weight data, this variable is captured in a validated province-wide database. Thus, we can speculate that the interobserver variability among NT and birth weight measurement is low and unlikely to affect our outcome.

In summary, we found a positive association between first trimester intertwin CRL difference greater than 10%, NT discordance (>14%), and absolutes NT difference (greater than 95^th^ percentile) with twin birth weight discrepancy. Furthermore, we show that CRL discordance greater than 10% and an absolute NT difference corresponding to the 95^th^ percentile are associated with an increased likelihood of twin birth weight discordance greater than 20%. Pregnancies with high CRL and NT discordance captured during the first trimester warrant careful surveillance to ensure healthy interval growth of each twin and patient counselling as per current guidelines.

## Figures and Tables

**Table 1:** Subject characteristics in twins with a birth weight discordance < 20% vs. ≥ 20%.

Characteristics (*n* = 887)	Birth weight discordance < 20% (*n* = 742)	Birth weight discordance ≥ 20% (*n* = 145)	*P* value
Maternal age, years (mean, SD)	32.7 (4.9)	33.2 (4.8)	0.3423
Maternal BMI (kg/m2) (*n*, %)			
(i) < 18.5	20 (3.01%)	3 (2.27%)	0.0818
(ii) 18.5-24.9	362 (54.44%)	74 (56.06%)	
(iii) 25-29.9	171 (25.71%)	23 (17.42%)	
(iv) 30+	112 (16.84)	32 (24.24%)	
Parity *n* (%)			
0	423 (58.34%)	90 (62.50%)	0.4281
1	222 (30.62%)	43 (29.86%)	
≥ 2	80 (11.03%)	11 (7.64%)	
IVF, *n* (%)			
Yes	171 (23.68%)	30 (20.98%)	0.4841
No	551 (76.32%)	113 (79.02%)	
CRL difference (mm, SD)	2.6 (2.9)	4.5 (4.4)	< 0.0001
CRL ratio (%)	0 (0)	0.1 (0.1)	< 0.0001
NT difference (mm)	0.3 (0.5)	0.4 (0.6)	0.1598
NT ratio (%)	0.1 (0.1)	0.2 (0.2)	0.0775
Average birth weight (twin 1 and 2, grams SD)	2445 (549)	2131 (638)	< .0001
Weight difference (grams, SD)	204 (155)	732 (284)	< .0001
Gestational age at delivery (weeks)	35.8 (2.6)	34.7 (3.0)	0.0002
Fetal sex			
Male:Male (%)	229 (31.46%)	52 (36.88%)	0.2793
Male:Female (%)	257 (35.30%)	51 (36.17%)	
Female:Female (%)	242 (33.24%)	38 (26.95%)	
Chorionicity, ultrasound (*n*, %)			
DCDA	547 (76.93%)	115 (82.14%)	0.1415
MCDA	149 (20.96%)	25 (17.86%)	
MCMA	15 (2.11%)	0 (0%)	
Chorionicity, pathology (*n*, %)			
DCDA	461 (77.61%)	97 (80.83%)	0.2451
MCDA	120 (20.20%)	23 (19.17%)	
MCMA	13 (2.19%)	0 (0%)	

Legend: SD: standard deviation; BMI: body mass index; IVF: in vitro fertilization; CRL: crown rump length; NT: nuchal translucency; DCDA: dichorionic diamniotic; MCDA: monochorionic diamniotic; MCMA: monochorionic monoamniotic.

**Table 2:** Multivariate probability modeling the effect of CRL discordance ≥ 10% on birth weight discordance.

Odds ratio estimates				
Effect	Point estimate	95% Wald confidence limits		*P* value
CRL discordance ≥ 10%	3.715	2.248	6.141	< .0001
Chorionicity via US MCDA vs. DCDA	0.860	0.516	1.432	0.3159
Chorionicity via US MCMA vs. DCDA	0.150	0.007	3.010	0.2335
Maternal age	1.002	0.961	1.044	0.9349
Gestational age at delivery	0.873	0.816	0.934	< .0001
Maternal BMI < 18.5 vs. 18.5-24.9	1.232	0.373	4.065	0.7375
Maternal BMI 25-29.9 vs. 18.5-24.9	0.727	0.433	1.222	0.1098
Maternal BMI 30+ vs. 18.5-24.9	1.399	0.841	2.328	0.2291
Parity 1 vs. 0	1.121	0.721	1.744	0.0671
Parity 2+ vs. 0	0.441	0.184	1.053	0.0454

**Table 3:** Multivariate probability modeling the effect of NT discordance ≥ 20% on birth weight discordance.

Odds ratio estimates				
Effect	Point estimate	95% Wald confidence limits		*P* value
NT discordance ≥ 20%	1.168	0.771	1.771	0.4641
Chorionicity via US MCDA vs. DCDA	0.846	0.513	1.394	0.2863
Chorionicity via US MCMA vs. DCDA	0.13	0.006	2.674	0.2044
Maternal age	1.005	0.964	1.047	0.8186
Gestational age at delivery	0.873	0.818	0.933	< .0001
Maternal BMI < 18.5 vs. 18.5-24.9	0.926	0.281	3.053	0.9411
Maternal BMI 25-29.9 vs. 18.5-24.9	0.706	0.424	1.173	0.188
Maternal BMI 30+ vs. 18.5-24.9	1.288	0.783	2.12	0.1988
Parity 1 vs. 0	0.971	0.629	1.5	0.1409
Parity 2+ vs. 0	0.41	0.174	0.969	0.0429

**Table 4:** Multivariate probability modeling the effect of NT discordance >/ = 14% on birth weight discordance.

Odds ratio estimates				
Effect	Point estimate	95% Wald confidence limits		*P* value
NT discordance ≥ 14%	1.086	0.734	1.606	0.6807
Chorionicity via US MCDA vs DCDA	0.847	0.514	1.395	0.2734
Chorionicity via US MCMA vs DCDA	0.125	0.006	2.548	0.1939
Maternal age	1.005	0.965	1.047	0.8092
Gestational age at delivery	0.872	0.817	0.932	< .0001
Maternal BMI < 18.5 vs. 18.5-24.9	0.937	0.284	3.091	0.9585
Maternal BMI 25-29.9 vs. 18.5-24.9	0.706	0.425	1.174	0.1871
Maternal BMI 30+ vs. 18.5-24.9	1.282	0.778	2.113	0.2086
Parity 1 vs. 0	0.969	0.627	1.495	0.14
Parity 2+ vs. 0	0.407	0.172	0.961	0.0414

**Table 5:** Multivariate probability modeling the effect of NT difference ≥ 0.9 mm (95^th^ percentile) on birth weight discordance.

Odds ratio estimates				
Effect	Point estimate	95% Wald confidence limits		*P* value
NT difference ≥0.9 mm	2.538	1.215	5.299	0.0132
Chorionicity via US MCDA vs DCDA	0.772	0.462	1.289	0.3454
Chorionicity via US MCMA vs DCDA	0.131	0.006	2.701	0.2174
Maternal age	1.004	0.964	1.046	0.8464
Gestational age at delivery	0.873	0.817	0.932	< .0001
Maternal BMI < 18.5 vs. 18.5-24.9	0.852	0.254	2.861	0.8377
Maternal BMI 25-29.9 vs. 18.5-24.9	0.702	0.421	1.169	0.2176
Maternal BMI 30+ vs. 18.5-24.9	1.287	0.78	2.123	0.1729
Parity 1 vs. 0	0.973	0.629	1.503	0.1506
Parity 2+ vs. 0	0.419	0.177	0.99	0.0481

## Data Availability

Parties interested in this retrospective dataset are encouraged to contact the corresponding author Dr. Karen Fung-Kee-Fung as it is not publicly available.
